# Feasibility of contrast-enhanced ultrasonography (CEUS) in evaluating renal microvascular perfusion in pediatric patients

**DOI:** 10.1186/s12880-022-00925-z

**Published:** 2022-11-11

**Authors:** Wei Zhang, Huiming Yi, Baohuan Cai, Yonghua He, Shi Huang, Yu Zhang

**Affiliations:** 1 Department of Medical Ultrasound, Tongji Hospital, Tongji Medical College, Huazhong University of Science and Technology, Wuhan City, Hubei Province China; 2 Department of Pediatrics, Tongji Hospital, Tongji Medical College, Huazhong University of Science and Technology, Wuhan City, 430030 Hubei Province China

**Keywords:** Contrast enhanced ultrasonography, Pediatric renal disease, Chronic renal disease, Microvascular perfusion

## Abstract

**Background:**

Changes in renal microvascular perfusion are involved in several kidney diseases. Contrast-enhanced ultrasonography (CEUS) quantitative analysis can enable the estimation of renal microvascular perfusion non-invasively. However, to date, few pediatric patients with renal disease have been subjected to CEUS quantitative analysis. This study aimed to explore the feasibility of CEUS in evaluating renal microvascular perfusion in pediatric patients and paving its way to clinical practice.

**Methods:**

Seventeen pediatric patients with chronic kidney disease (CKD) and five children without kidney disease were consecutively examined using CEUS. Quantitative analysis of CEUS images based on time-intensity curve (TIC) fittings was performed using specialized software. Quantitative parameters of wash-in microvascular blood flow, including A, k, B, and TtoPk, were generated from three regions of interest (ROIs) each in the cortex and medulla of each kidney.

**Results:**

CEUS was performed in all children successfully and safely without the use of sedatives. All parameters (A, B, k, and TtoPk) demonstrated no statistical differences among the three sampling ROIs in the renal cortex and medulla. All parameters (A, B, k, and TtoPk) showed no statistical differences between the left and right sides of kidneys both in cortices and medullas. Comparing with patients with CKD stage 3–5, both control group and patients with CKD stage 1–2 had significantly higher values of parameter A in the renal cortex (*p* = 0.025 and *p* = 0.031, respectively). In control group and patients stage 1–2, the values of parameters k in the renal cortices were significantly higher than that in the renal medullas, while in patients with CKD stage 3–5, parameter k showed no statistically significant differences between the renal cortex and medulla (*p* = 0.173).

**Conclusion:**

CEUS is safe and practicable in pediatric patients with chronic kidney disease. Renal microvascular perfusion estimated by CEUS could be a robust approach in the evaluation of pediatric renal diseases. Parameters A and k derived from CEUS quantitative analysis can provide great potential in non-invasive assessment of renal microvascular perfusion impairment in pediatric CKD.

## Background

Changes in renal microvascular perfusion are associated with several kidney diseases, such as hypermicrovascular perfusion in diabetic kidney disease and reduced renal blood flow in acute kidney injury (AKI) [1–3]. Abnormalities in renal microvascular perfusion are associated with renal dysfunction and progressive alterations in the renal parenchyma [1, 3–8]. In addition, the degree of renal microvascular perfusion impairment can predict the severity of renal fibrosis [8]. Therefore, monitoring of renal microvascular perfusion could be a critical approach to detect early renal injury and subsequent progression of developing chronic kidney disease (CKD) [1, 3–9]. Moreover, these findings have delivered new insights into the management of renal diseases in clinical practice, as early identification of patients at high risks for CKD progression would be beneficial in planning appropriate renal-preserving treatments [8].

Contrast-enhanced computed tomography (CECT) and magnetic resonance imaging (CEMRI) have been used to measure tissue microvascular perfusion with intravenous injections of iodinated- and gadolinium-based contrast agents, respectively [10, 11]. As the contrast agents used in CECT and CEMRI techniques are predominantly excreted by kidneys, CECT and CEMRI are hazardous to patients with renal insufficiency [10–12]. Although functional MRI can be performed without the use of contrast agents, the lack of standardized sequences, post-processing software, and models hinder its routine clinical application [13].

Contrast-enhanced ultrasonography (CEUS) is a competing technology that involves the use of ultrasound contrast agents (UCAs) and specialized imaging techniques to enable real-time observation of tissue microvascular perfusion non-invasively [14]. UCAs are typically gas-filled microbubbles (MBs) encapsulated by a shell, usually comprised of phospholipid or albumin [14]. UCAs, which are similar in size to red blood cells, can resonate within the ultrasound imaging frequency to generate much stronger backscattering signals than red blood cells [14]. Since UCAs are strictly intravascular blood pool agents, they do not diffuse outside of the vessel except for active bleeding [14]. Therefore, the circulation of UCAs can emulate the rheology of red blood cells and allows for continuous imaging of the vasculature and blood flow [15]. Additionally, dynamic CEUS can provide quantitative parameters to estimate tissue microvascular perfusion [16]. Since the gas and shell of UCAs are cleared by the lungs and the reticuloendothelial system, respectively, UCAs are generally safe and allows for rapid applications of CEUS without laboratory testing for renal or hepatic functions [14, 15]. In summary, CEUS has significant advantages over other imaging modalities in the evaluation of renal microvascular perfusion, especially in patients with renal insufficiency.

Previously, several pre-clinical and clinical studies testing the application of CEUS in the evaluation of renal microvascular perfusion have shown promising results [17]. However, to the best of our knowledge, few pediatric patients has been involved in previous studies on the use of CEUS to assess renal microvascular perfusion[18]. In this study, we aimed to explore the feasibility of CEUS in evaluating renal microvascular perfusion in pediatric patients and to pave the way for its use in clinics. This study demonstrated the safety, reproducibility, and preliminary clinical prospect of CEUS quantitative analysis in pediatric patients with CKD.

## Materials and methods

Between February and June 2021, seventeen pediatric patients with CKD and five children without kidney disease were consecutively examined at our department using CEUS. Patients with hydronephrosis, renal artery stenosis, and congenital renal malformations were excluded from this study. The study was approved by the Institutional Review Board of Tongji Hospital and was performed in compliance with the principles of the World Medical Association Declaration of Helsinki, revised in 2000, Edinburgh. Written informed consent was obtained from a parent of each child prior to CEUS imaging.

### CEUS and imaging analysis

All ultrasound examinations were performed by the same radiologist (W. Z.) using the GE Logiq E9 Ultrasound system (GE Healthcare, Little Chalfont, UK). Without sedation, the patients were placed in the prone position.

Before CEUS, both kidneys were imaged in the longitudinal section at the position of renal hilum, and the maximum craniocaudal diameter was measured. The blood flow and pulsed-wave spectrum of the interlobar artery in each kidney were recorded using Doppler ultrasonography.

SonoVue (Barcco, Milan, Italy) was used as the contrast agent in this study. Each vial of contrast agent (containing 25 mg of lyophilized SonoVue) was reconstituted by adding 5 mL of saline (0.9% NaCl) solution and shaken evenly following the manufacturer’s recommendations. A bolus of the reconstituted suspension (0.03 mL/kg) was rapidly infused via an indwelling catheter placed in the upper limb vein, followed by flushing with 5 mL of the saline solution. While the contrast agent was injected, activation of a timer and CEUS imaging capture were initiated simultaneously. Kidneys were examined in the longitudinal plane at the position of the hilum, and CEUS was performed in the harmonic imaging mode, with a transducer frequency of 4 MHz, a depth of 4 cm, a mechanical index of 0.08, and a frame rate of 9 per second. Dynamic enhanced renal images were captured as a movie clip for 3 min. Each patient received two bolus injections of SonoVue for both kidneys separately, and the second bolus was injected approximately 10 min after the first bolus to ensure the clearance of circulating microbubbles. All system settings were identical and set fixed for all patients during this study.

**Fig. 1:**
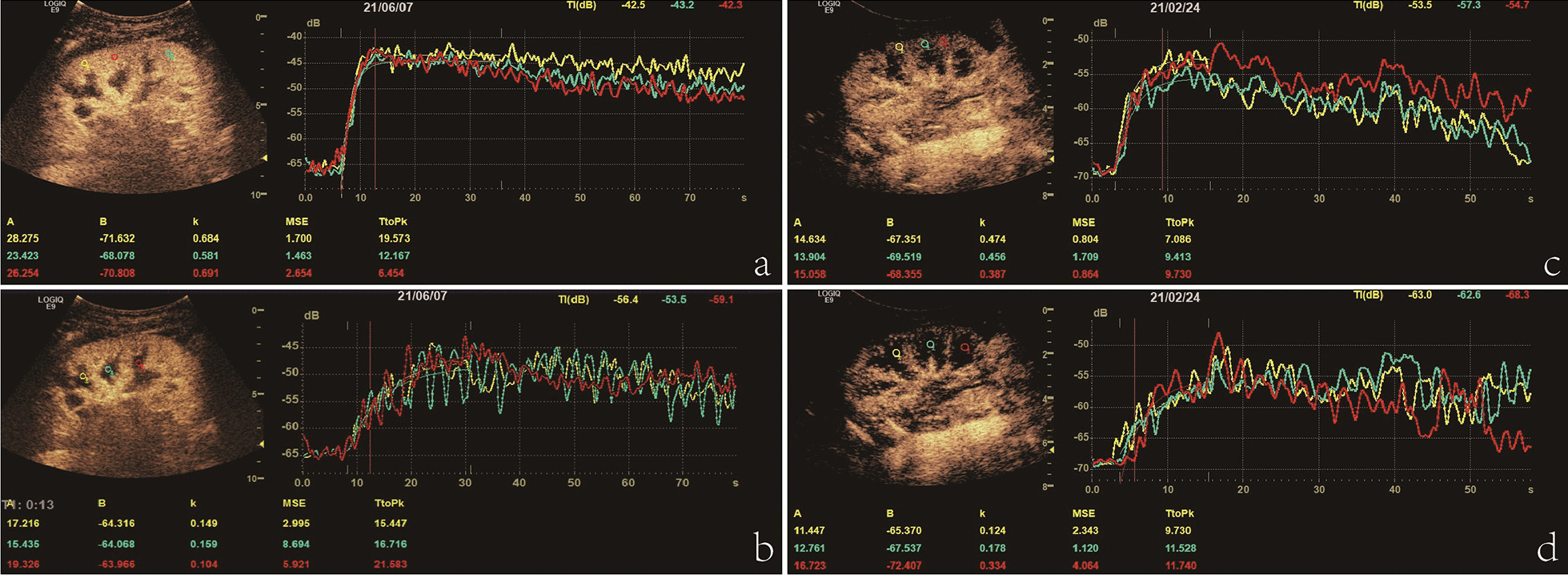
Quantitative analysis of contrast-enhanced ultrasonography (CEUS) images based on time-intensity curve fittings in a child without kidney disease (**a** &** b**) and in a pediatric patient with CKD stage 5 (**c** &** d**). Three regions of interest (ROIs) were placed in the cortices, and three ROIs were placed in the medullas. Quantitative parameters of wash-in blood flow, including A, B, k, and TtoPk, were generated automatically for each ROI

Quantitative analysis of CEUS images was based on time-intensity curve (TIC) fittings using specialized software equipped with GE Logiq E9 system [19]. Six regions of interest (ROIs) with identical areas were manually set: three in the renal cortex and three in the renal medulla, which were placed at approximately the same depth separately (Fig. 1). Quantitative parameters of wash-in blood flow were generated automatically from the TIC fitting formula.

1 $$\text{F}\left(\text{t}\right)=\text{A}\left(1-\text{exp}\left[-\text{k}\text{t}\right]\right)+\text{B})$$

where the following TIC quantitative parameters were involved: [1] A, the plateau value as an estimate of the regional blood volume; [2] B, the baseline; [3] k, the slope of the ascending TIC as an estimate of mean blood velocity; [4] time to peak intensity (TtoPk), defined as the time from the start of enhancement to the time when contrast agent microvascular perfusion reached the peak [19]. All parameters were acquired by the same investigator (HM. Y.)

### Statistical analysis

All quantitative data are expressed as the mean ± standard deviation (SD). Non-parametrical tests were used due to the small number of children and non-normally distributed data involved in this study. Differences of parameters among the three ROIs in the renal cortex and in the renal medulla were investigated using Kruskal-Wallis tests. The mean values of parameters from the three sampling ROIs in the renal cortex and medulla were used for subsequent statistical analyses. Parameters between left and right sides of kidneys as well as between renal cortices and renal medullas were compared using Wilcoxon tests. Mann-Whitney tests were used to compared the parameters between patients with different CKD stages. For all tests, values with p < 0.05 were considered statistically significant.

## Results

A total of seventeen pediatric patients with CKD were enrolled in this study, of which, one patient with stage 5 (eGFR 4.5 ml/min), two patients with stage 3 (mean eGFR 54.4 ml/min), four patients with stage 2 (mean eGFR 73.8 ml/min) and ten patients with stage 1 (mean eGFR 121.3 ml/min). In addition, five pediatric patients without kidney disease (mean eGFR 119.6 ml/min) were also included in this study as a control group. Of these patients, nine were females and thirteen were males, with a mean age of 8.4 ± 3.2 years. The CEUS procedures were performed in all pediatric patients successfully and safely, and no side effects were observed regardless of eGFR value. After bolus injection of the contrast agent, the renal cortex was enhanced initially followed by a more gradual enhancement of the medulla. No side effects were observed in any of the patients during and post CEUS in this study. The clinical features of these patients are presented in Table 1.

**Table 1:** Clinical features of pediatric patients included in this study

No	Sex	Age (years)	CKD stage	Left kidney			Righ kidney			Primary diagnosis
				LD	SD	RI	LD	SD	RI	
1	Male	6.5	CKD 0	8.4	4.2	0.62	8.3	4.2	0.62	None
2	Male	10.3	CKD 0	9.1	4	0.64	9	4	0.67	None
3	Female	2.4	CKD 0	7.4	3.2	0.68	7.4	3.4	0.69	None
4	Female	6.7	CKD 1	7.8	3.6	0.67	7.8	3.7	0.65	Nephrotic syndrome
5	Female	11.7	CKD 1	10.2	3.9	0.68	9.7	3.7	0.66	Nephrotic syndrome
6	Male	8.7	CKD 1	8.8	4.3	0.59	8.9	4.4	0.63	Nephrotic syndrome
7	Male	1.6	CKD 1	5.7	2.7	0.85	6.1	3	0.89	Hypercalciuria
8	Male	10.7	CKD 1	9.3	4.6	0.63	9.8	4	0.62	Alport syndrome
9	Female	9.1	CKD 1	8.4	4.1	0.61	8.2	4.2	0.61	IgA nephropathy
10	Female	8.5	CKD 1	9.2	4	0.6	9.9	4.4	0.6	HSPN
11	Female	9.3	CKD 1	8.5	3.8	0.62	8.3	4.2	0.56	HSPN
12	Male	6.5	CKD 1	8.4	3.9	0.73	8.5	3.8	0.74	IgM nephropathy
13	Female	10.4	CKD 1	9.4	4.1	0.66	9.6	3.9	0.66	IgA nephropathy
14	Male	10.2	CKD 1	9.5	4.4	0.6	9.1	4.9	0.56	IgA nephropathy
15	Female	11.1	CKD 1	9.8	4.1	0.65	10.4	4.2	0.68	IgA nephropathy
16	Male	10.7	CKD 2	9.2	4.2	0.67	9.8	4.5	0.63	IgA nephropathy
17	Male	13.2	CKD 2	10.8	4.6	0.63	10.5	4.5	0.6	Lupus nephritis
18	Male	11.3	CKD 2	9.9	4.9	0.76	9.8	4.8	0.69	IgA nephropathy
19	Female	1.3	CKD 2	7.7	3.3	0.67	7.6	3.2	0.66	MPGN
20	Male	7	CKD 3	7.3	2.2	0.72	8	2.5	0.71	Chronic interstitial nephritis
21	Male	10.9	CKD 3	7.6	3.5	0.61	8	3.3	0.58	Primary hyperoxaluria type 1
22	Male	5.7	CKD 5	7	2.8	0.63	6.7	2.4	0.64	Urinary system infection

*LD* long-axis diameter (cm), *SD* short-axis diameter (cm), *RI* resistance index of interlobar artery

*MPGN* Membranoproliferative glomerulonephritis, *HSPN* Henoch-Schonlein purpura nephritis

### Reproducibility of TIC parameters in the renal cortex and medulla (Fig. 2)

**Fig. 2:**
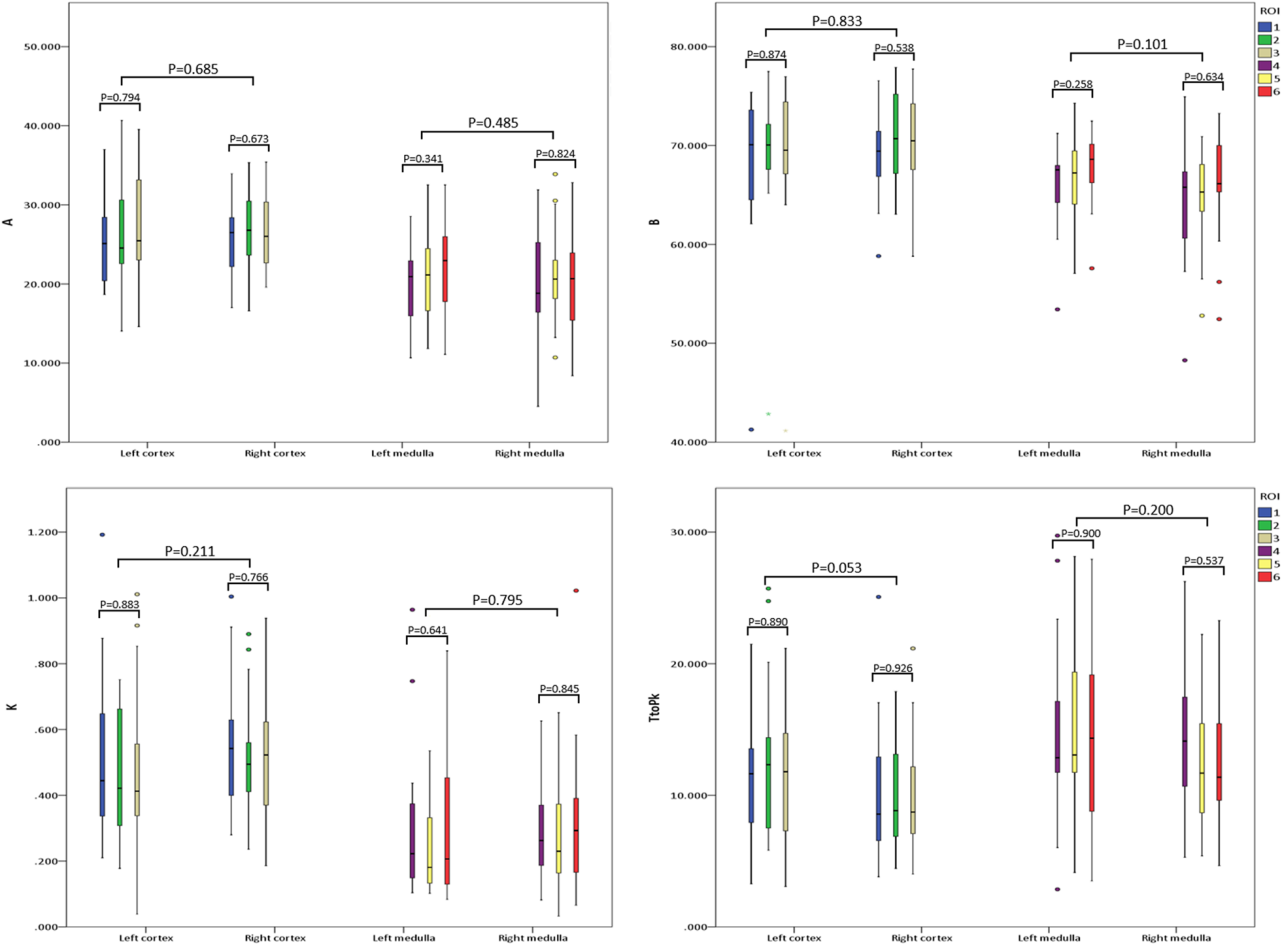
Statistical analyses of contrast-enhanced ultrasonography (CEUS)-derived quantitative parameters (A, B, k, and TtoPk) between the renal cortex and medulla as well as the left and right kidneys. Regions of interest (ROIs) 1–3 are located in the renal cortex, and ROIs 4–6 are located in the renal medulla. These results showed robust reproducibility of CEUS quantitative parameters in analyzing microvascular perfusion in pediatric kidneys

When analyzing the renal cortex on the same kidney (left or right), no statistically significant differences were observed among the three ROIs in terms of parameters A (*p* = 0.794 for the left kidney and *p* =  0.673 for the right kidney), B (*p* = 0.874 for the left kidney and  *p* = 0.538 for the right kidney), k (*p * = 0.883 for the left kidney and  *p* = 0.766 for the right kidney), and TtoPk (*p *= 0.890 for the left kidney and * p * = 0.926 for the right kidney). In addition, there were no statistically significant differences between the left and right renal cortices in terms of parameters A (*p * = 0.685), B (*p * = 0.833), k (*p * = 0.211) and TtoPk (*p * = 0.038).

While comparing the renal medulla in the same kidney (left or right), there were no statistically significant differences among the three ROIs in terms of parameters A (*p * = 0.341 for the left kidney and  *p* = 0.824 for the right kidney), B (*p * = 0.258 for the left kidney and  *p * = 0.634 for the right kidney), k (*p * = 0.641 for the left kidney and *p * = 0.845 for the right kidney), and TtoPk (*p * = 0.900 for the left kidney and *p * = 0.537 for the right kidney). Moreover, no statistically significant differences were observed between the left and right renal medullas in terms of parameters A (*p * = 0.485), B (*p * = 0.101), k (*p *= 0.795) and TtoPk (*p * = 0.200).

### **Parameter of TICs among patients with CKD stage 3–5, patients with CKD stage 1–2 and control group (**Fig. 3**)**

**Fig. 3:**
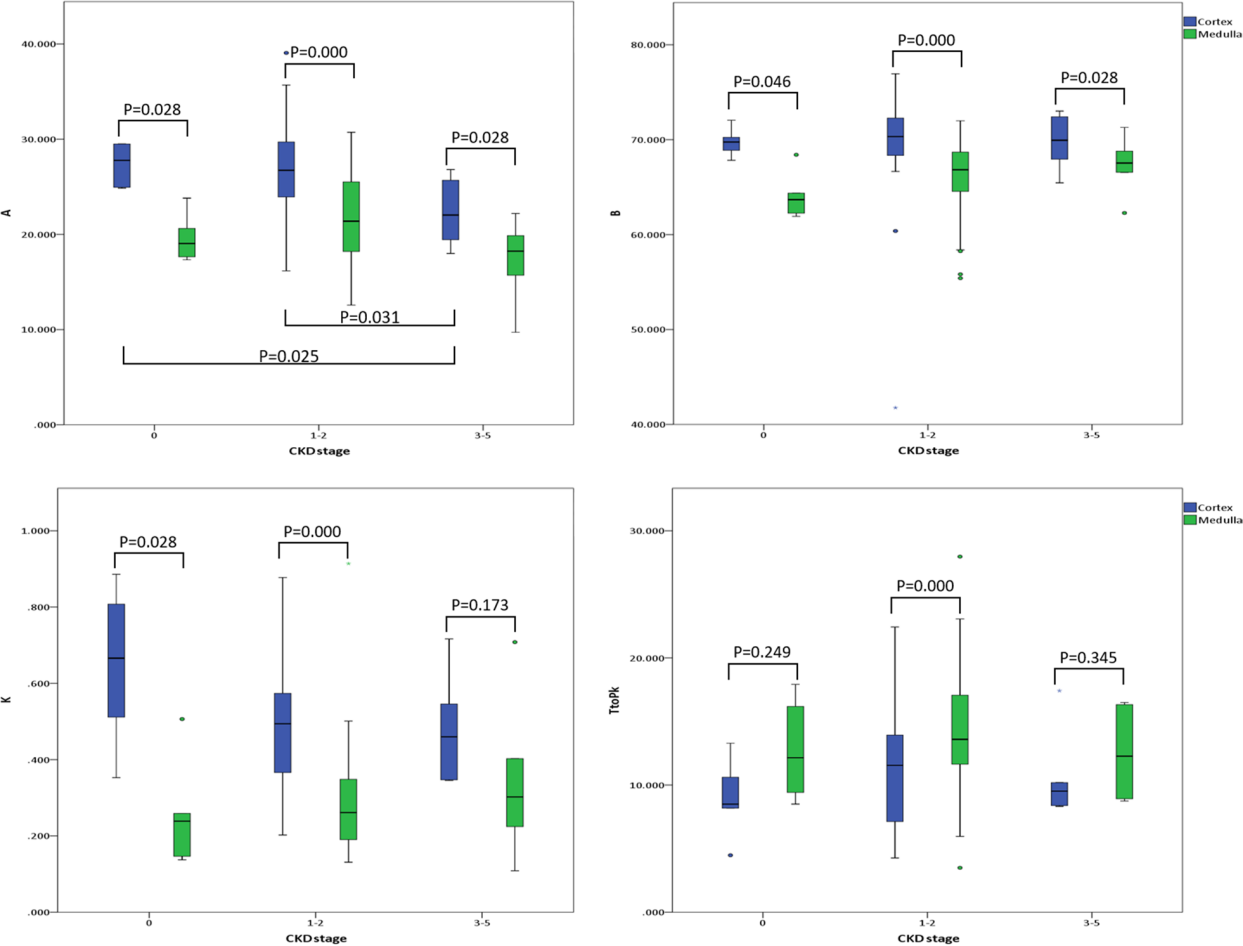
Statistical analyses of contrast-enhanced ultrasonography (CEUS)-derived quantitative parameters (A, B, k, and TtoPk) in the renal cortex and medulla between patients with CKD stage 3–5 and those with CKD stage 1–2. These results showed the potential of CEUS quantitative analysis in evaluating pediatric renal diseases

As twenty-two patients mentioned above were included in this study, a total of forty-four kidneys (six from control group, thirty-two from CKD stage 1–2, and six from CKD stage 3–5) were analyzed.

Comparing with patients with CKD stage 3–5, both control group and patients CKD stage 1–2 had significantly higher values of A in the renal cortex (*p* = 0.025 and *p* = 0.031, respectively). However, there was no statistically significant difference between control group and patients with stage 1–2 in terms of parameter A in the renal cortex.

When comparing the kidneys among patients with CKD stage 3–5, CKD stage 1–2 and control group, the values of parameter A showed no statistical significant differences in the renal medulla.

Additionally, in the renal cortex, no statistically significant differences were observed among control group, patients with CKD stage 1–2 and patients with CKD stage 3–5 in terms of parameters B, k and TtoPk. Meanwhile, in the renal medulla, no statistically significant differences existed among these three groups in terms of parameters B, k and TtoPk.

### Parameters of TICs between the renal cortex and medulla (Fig. 3)

In control group, the renal cortex had significantly higher values than the medulla in terms of parameters A (*p* = 0.028), B (*p* = 0.046), and k (*p* = 0.028). Although the values of parameter TtoPk in the renal cortex were lower than that in the medulla, the statistically significant difference was not observed (p = 0.249).

In patients with CKD stage 1–2, when compared with the medulla, the cortex had significantly higher values in terms of parameters A (*p* = 0.000), B (*p* = 0.000), k (*p* = 0.000) and TtoPk (*p * =  0.000).

In patients with CKD stage 3–5, the values of parameters A and B in the renal cortex were significantly higher than those in the medulla (*p* = 0.028 and *p* = 0.028, respectively). However, there were no statistically significant differences between the renal cortex and medulla in terms of parameters k (*p*= 0.173) and TtoPk (*p* = 0.345).

## Discussion

The present study demonstrated the feasibility of CEUS in pediatric kidneys. At present, two main approaches are used to estimate tissue microvascular perfusion using CEUS: bolus kinetics and flash-replenishment kinetics. For the bolus method, a bolus of ultrasound contrast agents is administered intravenously, and a curve representing the acoustic intensity over time is obtained, while flash-replenishment kinetics is based on the reappearance of enhancement after the complete destruction of the microbubbles by a flash of high mechanical intensity during continuous intravenous infusion of contrast agents [16]. As the bolus method is more convenient for routine clinical practice, this study was performed based on bolus kinetics. CEUS was safely and successfully performed in all children enrolled in this study. In addition, although the children were not sedated and renal motions existed naturally, quantitative parameters were obtained from TIC analyses. Previously, histological assessments in porcine models demonstrated that ultrasound contrast agents and the CEUS procedures did not induce any tissue damage to the kidneys [20]. Therefore, this study suggests that CEUS is safe for routine practice in pediatric patients with renal diseases.

This study revealed the reproducibility of CEUS quantitative analysis in pediatric renal diseases. The major challenge in the widespread clinical uptake of CEUS quantitative analysis is the relatively high degree of variability, which may cause diagnostic uncertainty [16, 21]. This variability is related to several factors including the operator, machine settings, the contrast agent, and physiologic patient factors [16]. To minimize the variations due to the abovementioned factors, this study was implemented according to a standardized protocol. Image acquisition and data processing were performed by the same operator separately, who made sure that the machine settings, the imaging planes, bolus injection procedures, ROI placements, and quantification processes were comparable for each kidney. Moreover, to eliminate the variations in the examination of both of the kidney, the present study compared the renal cortex and medulla in the same kidney, and values of the TIC-derived parameters (A, B, k, and TtoPk) demonstrated no statistical differences among the three sampling ROIs in the renal cortex and medulla. When comparing left and right kidneys, values of all TIC-derived parameters (A, B, k, and TtoPk) showed no statistical differences between the left and right kidneys both in renal cortices and renal medullas. These results verified the reproducibility of CEUS quantitative analysis in pediatric kidneys. Pre-clinical studies have reported that CEUS-derived parameters were comparable to absolute measurements of blood flow in rat kidneys [22]. Additionally, the location and size of ROIs did not significantly affect the renal microvascular perfusion metrics evaluated by CEUS in dogs [23]. Therefore, this study suggests that CEUS quantitative analysis could be a robust tool to evaluate renal microvascular perfusion in children.

This study demonstrated the possibility of using CEUS-derived parameters in differentiating different stages of CKD. When comparing the kidneys among patients with CKD stage 3–5, patients with CKD stage 1–2 and control group, values of parameter A were significantly lower in CKD stage 3–5 than in stage 1–2 and control group in the renal cortex. In addition, parameter A showed no significant difference in the renal cortex between patients with CKD stage 1–2 and control group. Moreover, in both the renal cortex and medulla, no statistically significant differences were observed among control group, patients with CKD stage and patient with CKD stage 3–5 in terms of parameters B, k and TtoPk. These results indicate that parameter A in the renal cortex could provide great potential in non-invasive assessment of renal microvascular perfusion impairment in pediatric CKD, which is in agreement with previous studies on evaluation of renal microvascular perfusion in adult humans; those studies suggested that CEUS enables the monitoring of renal injury in both acute and chronic renal diseases [1, 3–7].

Additionally, this study compared the CEUS-derived parameters between the renal cortex and medulla. In patients with various CKD stages, values of parameters A and B were all significantly lower in renal medulla than in the cortex. These differences in the values of the examined parameters between the cortex and medulla may be related to the specific physiologic characteristics of the medullary blood supply, which only receives 10% of the total renal blood flow [24]. Furthermore, in control group and patients with CKD stage 1–2, the values of parameter k were significantly higher in the cortices than in the medullas, and the values of parameter TtoPk were significantly lower in the cortices and in the medullas. However, the values of parameter k and TtoPk showed no significant differences between renal cortex and medulla in patients with CKD stage 3–5. The discrepancies may result from impaired hemodynamics in the cortical and medulla blood supply as CKD progresses.

In summary, based on four parameters (A, B, k and TtoPk) derived from CEUS quantitative analysis, the reproducibility of CEUS in evaluating renal microvascular perfusion in pediatric patients was verified in this study. Among these parameters, A and k can serve as an estimation of regional blood volume and mean blood velocity, respectively [19]. Furthermore, this study showed that, with the progression of pediatric CKD, the value of parameter A in the renal cortex decreased, and the difference of the value of parameter k between renal cortex and medulla also tended to decrease. These findings showed that CEUS quantitative analysis revealed the reduction in blood volume and blood velocity in the renal cortex with the progression of CKD, while no microvascular hemodynamic differences in the renal medulla were detected. Based on these preliminary findings, repeated CEUS analysis at the time of diagnosis and at regular intervals during treatment may benefit pediatric patients with CKD through monitoring of renal microvascular perfusion and timely prediction of treatment outcomes.

This study has several limitations. First, the results have to be interpreted with caution due to the small number of pediatric patients involved, and further prospective studies with larger cases are required to refine the findings. Second, tissue motions, which are inevitable during CEUS in conscious pediatric patients, lead to data variations in quantitative analysis; nonetheless, this study suggests that CEUS quantitative analysis can still provide meaningful results by implementing a standard protocol. Third, all ROIs were placed manually on ultrasound images, but it is impossible to strictly delineate the outer and inner medulla, which have different blood flow dynamics. This may cause instability of statistical results involving the medulla. Fourth, since this is a preliminary study on the feasibility of CEUS quantitative analysis in pediatric renal diseases, only imaging-derived parameters were analyzed. Because of the small number of cases, we did not analyze the relationships between CEUS-derived parameters and various clinical indices; however, we plan to analyze them in our further studies.

## Conclusion

CEUS is a safe and practicable imaging technique for the evaluation of pediatric patients with renal disease. Renal microvascular perfusion estimated by CEUS could be a robust approach in the evaluation of pediatric renal diseases. Parameters A and k derived from CEUS quantitative analysis can provide great potential in non-invasive assessment of renal microvascular perfusion impairment in pediatric CKD. This study lays the foundation for further systematic evaluation of CEUS quantitative analysis in pediatric renal diseases in routine clinical practice.

## Data Availability

The datasets used and analyzed during this study are available from the corresponding author on reasonable request.

## Abbreviations

CEUS: Contrast-enhanced ultrasonography
TIC: Time-intensity curve
ROIs: Regions of interest
MBs: Microbubbles
UCAs: Ultrasound contrast agents
CKD: Chronic kidney disease
AKI: Acute kidney injury
CECT: Contrast-enhanced computed tomography
CEMRI: Contrast-enhanced magnetic resonance imaging
